# Targeting the Epidermal Growth Factor Receptor Can Counteract the Inhibition of Natural Killer Cell Function Exerted by Colorectal Tumor-Associated Fibroblasts

**DOI:** 10.3389/fimmu.2018.01150

**Published:** 2018-05-29

**Authors:** Delfina Costa, Roberta Venè, Roberto Benelli, Emanuele Romairone, Stefano Scabini, Silvia Catellani, Barbara Rebesco, Luca Mastracci, Federica Grillo, Simona Minghelli, Fabrizio Loiacono, Maria Raffaella Zocchi, Alessandro Poggi

**Affiliations:** ^1^Molecular Oncology and Angiogenesis Unit, Ospedale Policlinico San Martino, Genoa, Italy; ^2^Immunology Unit, Ospedale Policlinico San Martino, Genoa, Italy; ^3^Oncological Surgery, Ospedale Policlinico San Martino, Genoa, Italy; ^4^Clinical Hematology, Ospedale Policlinico San Martino, Genoa, Italy; ^5^Antiblastic Drug Unit, Ospedale Policlinico San Martino, Genoa, Italy; ^6^Unit of Pathology, Ospedale Policlinico San Martino, Genoa, Italy; ^7^Clinical and Experimental Immunology Laboratory, Ospedale G. Gaslini, Genoa, Italy; ^8^Division of Immunology, Transplants and Infectious Diseases, San Raffaele Scientific Institute, Milan, Italy

**Keywords:** CRC, NKG2D, natural killer cells, EGFR, antibody-dependent cellular cytotoxicity, cetuximab, TME

## Abstract

Mesenchymal stromal cells (MSC) present in the tumor microenvironment [usually named tumor-associated fibroblasts (TAF)] can exert immunosuppressive effects on T and natural killer (NK) lymphocytes, favoring tumor immune escape. We have analyzed this mechanism in colorectal carcinoma (CRC) and found that co-culture of NK cells with TAF can prevent the IL-2-mediated NKG2D upregulation. This leads to the impairment of NKG2D-mediated recognition of CRC cells, sparing the NK cell activation through DNAM1 or FcγRIIIA (CD16). *In situ*, TAF express detectable levels of epidermal growth factor receptor (EGFR); thus, the therapeutic anti-EGFR humanized antibody cetuximab can trigger the antibody-dependent cellular cytotoxicity of TAF, through the engagement of FcγRIIIA on NK cells. Importantly, in the tumor, we found a lymphoid infiltrate containing NKp46^+^CD3^−^ NK cells, enriched in CD16^+^ cells. This population, sorted and cultured with IL-2, could be triggered *via* CD16 and *via* NKG2D. Of note, *ex vivo* NKp46^+^CD3^−^ cells were able to kill autologous TAF; *in vivo*, this might represent a control mechanism to reduce TAF-mediated regulatory effect on NK cell function. Altogether, these findings suggest that MSC from the neoplastic mucosa (TAF) of CRC patients can downregulate the immune cell recognition of CRC tumor cells. This immunosuppression can be relieved by the anti-EGFR antibody used in CRC immunotherapy.

## Introduction

Several reports have shown that mesenchymal stromal cells (MSC) can exert potent immunosuppressive effects leading to the downregulation of T and natural killer (NK) cell responses; indeed, the interaction between MSC isolated from bone marrow and effector lymphocytes can lead to the suppression of T cell proliferation and NK cell cytotoxic activity (1–7). In addition, MSC-mediated immunosuppression has been reported to favor tumor growth in animal models (8). These cells are represented in the tumor microenvironment as tumor-associated fibroblasts (TAF), myofibroblasts, pericytes, adipocytes, lymphatic, and vascular endothelial cells (9, 10). All these cell types can influence the antitumor immune response and the fate of tumor cells (9–12). In normal gut, MSC are a major structural component of crypts, constituting the peri-cryptal fibroblast sheath (13). The intestinal crypt is a proliferative compartment maintained by stem cells and some evidences support that colorectal carcinoma (CRC) can emerge at the niche of tissue stem cells (14). Thus, MSC can play a role in regulating epithelial cell proliferation and differentiation (13–15); more importantly, some reports have indicated MSC as an attractive target for antitumor vaccine (16–19). Recently, it has been shown in a murine model that targeting TAF and tumor cells is effective in modifying tumor microenvironment. Indeed, the use of tumor cells expressing fibroblast activation protein as a vaccine resulted in the elimination of solid tumor and impairment of vascular dissemination. Importantly, this effect was accompanied by tumor infiltration of CD8^+^ T cells, reduction of TAF number and inhibition of the recruitment of immunosuppressive cells within the tumor (16–19). Based on these considerations, it is relevant to determine whether MSC derived from CRC mucosa display immunosuppressive properties. Indeed, it has been shown that TAF in melanoma patients may exert potent inhibiting effects on NK cell responses (20). In addition, TAF from head and neck cancer can inhibit T cell proliferation, further suggesting that mesenchymal cells can regulate the antitumor immune response (21). These findings indicate that also in humans MSC can be a suitable target for cancer therapies aimed to revert their immunosuppressive effect and allow the development of NK cell-mediated antitumor response (9). NK cells can bind to tumor targets through the interaction of lymphocyte function associated (LFA)1 and intercellular adhesion molecule (ICAM)1 expressed on tumor cells (22). This interaction leads to the engagement of NK cell activating receptors, including NKG2D or DNAM1 (23, 24), which bind to their ligands on tumor cells triggering their killing (23–25). In particular, CRC cells can express the NKG2D ligands (NKG2D-L) MICA and ULBPs, or the DNAM1 ligands (DNAM1-L) poliovirus receptor (PVR) and nectin-2: NKG2D–NKG2D-L and DNAM1–DNAM1-L interaction would lead to CRC cell recognition and killing by NK effector cells (26). NK cells can kill tumor cells also by the triggering of antibody-dependent cellular cytotoxicity (ADCC) through the engagement of FcγRIIIA (CD16) (27). This mechanism has been exploited in anti-cancer immunotherapy with the anti-epidermal growth factor receptor (EGFR) antibody cetuximab in KRAS wild type CRC (27, 28). Thus, the presence of functional NK cells can contribute both to CRC cell control and to the success of immunotherapy.

In this work, we show that: (1) TAF downregulate the NKG2D-mediated killing of CRC and prevent the IL-2 induced NKG2D upregulation on NK cells. (2) TAF express EGFR and can be killed by NK cells in ADCC triggered *via* the anti-EGFR antibody cetuximab. (3) NKp46^+^CD3^−^ NK cells found at the tumor site, sorted and cultured with IL-2, can kill autologous TAF.

## Materials and Methods

### Monoclonal Antibodies (mAbs) and Reagents

Anti-NKG2D (MAB139, IgG1), anti-DNAM1 (MAB666, IgG1), anti-CD32 (MAB1330, IgG2a), anti-CD64 (FAP12571, IgG1), anti-CD56 (301040, IgG2b), anti-CD90 (FAB2067p, IgG2a), anti-PVR (MAB25301, IgG1), anti-ULBP1 (MAB1380, IgG2a), ULBP2 (MAB1298, IgG2a), ULBP3 (MAB15171, IgG2a), and anti-CD146 (MAB932, IgG1) mAbs were purchased from R&D System (Minneapolis, MN, USA). The anti-CD3 mAb (JT3A, IgG2a), the anti-CD16 mAbs (NK1, IgG1 and NK54, IgG2a), the anti-CD18 mAb (70H12, IgG2a), the anti-CD54 mAb (ICAM1, clone SM89, IgM), the anti-MICA (M320, IgM), and the anti-CD45 (T205, IgM) were obtained in our laboratory (4). The PE-anti-NKp46 (9E2, IgG1) was purchased from Miltenyi biotech (Germany, EU); Alexafluor488-anti-CD45 (HI30, IgG1), PE-Cy7-anti-NKp46 (9E2, IgG1), PE/Dazzle-anti-CD3 (UCHT1, IgG1), PE-Cy5 anti-CD56, Pacific Blue-anti-CD16, and anti-NKG2A (16A11, IgG2b) mAbs were from BioLegend (San Diego, CA, USA). The anti-SH2 (CD105, IgG1), the anti-SH3 (CD73, IgG2b), the anti-CD11a (LFA1α, TS1.22, IgG1), and the anti-CD18 (LFA1β, TS1.18, IgG1) producing hybridomas were purchased from the American Type Culture Collection (Manassas, VA, USA). Anti-vimentin mAb was from Dako Cytomation (clone V9) and anti-collagen I was from Novus Biologicals LLC (Littelton, CO, USA, clone NB600-450). The therapeutic anti-EGFR cetuximab and anti-CD20 rituximab humanized antibodies were from the Antiblastic Drug Unit of the Policlinico San Martino (Genoa, Italy) as the leftover of the dose delivered to patients for immunotherapy. Complete medium was composed of RPMI1640 (Thermo Fisher Scientific, Carlsbad, CA, USA) with 10% of fetal calf serum (FCS, Sigma) supplemented with 1% antibiotics (penicillin and streptomycin) and 1% l-glutamine (Thermo Fisher Scientific).

### CRC Cell Lines and Cell Isolation From Tumor Specimens

CRC cell lines Caco2, HT29, HCT15, SW480, DLD1, HCT116, LS180, WiDr, LoVo, Colo205, Colo320 DMF, SW620, T84, and SW48 were from cell bank of the IRCCS (kind gift of Blood Transfusion Centre, Dr. Barbara Parodi), and for the three CRC cell lines used in cytolytic assays (Caco2, HCT15, and SW480), the correct identification was determined with STR DNA profile. Peripheral blood mononuclear cells (PBMC) were obtained from venous blood samples of healthy donors of the Transfusional Centre of the Policlinico San Martino (upon signed institutional informed consent, according to a procedure defined by the Regional Ethic Committee, CONSAZH780148/17/07/2015) after Ficoll-Hypaque density gradient separation (29). NK cells from PBMC, or from tumor tissue suspensions (see below), were obtained using the corresponding RosetteSep negative separation kit (StemCell Technologies SARL, Grenoble, France). The purity of NK cell populations ranged from 75 to 95% for the expression of CD56, 65 to 85% CD16^+^, and >99.8% of CD3^−^ cells. The CD3^−^ but CD56^−^ and CD16^−^ cells were CD20^+^CD19^+^ B cells (5–15%). CRC specimens were obtained from the Surgical Oncology Unit during therapeutic surgery after signed informed consent (Institutional and Regional Ethic Committee approval, PR163REG2014). Characterization of NK phenotype in cell suspensions, obtained as described (30), from tumor specimens or macroscopically tumor free mucosa counterpart, was performed by FACS analysis. In some experiments, NK cells were separated from tumor cell suspensions (*n* = 5), by FACS sorting (FACSAria II, BD Biosciences, San Josè, CA, USA) after double staining with PE-Cy7-anti-NKp46 and PE/Dazzle-anti-CD3 mAbs. Purity of cell sorting was 98.5% (two anti-CD3 mAbs, JT3A and UCHT1, were used to define the purity of the bulk population as CD3^−^ NK cells). Cells were then put in culture in RPMI1640 supplemented with 10% FCS and 10 ng/ml IL-2 (30 IU/ml, Miltenyi Biotech GmbH, Bergisch, Gadbach, Germany, specific activity 3 × 10^6^IU) on irradiated PBMC as feeder cells in U-bottomed plates. Cell cultures were then split when evident cell growth was microscopically detected, checked for the expression of NKP46 on day 25 (>90%) and used in functional assays. Generation of primary MSC lines was obtained after mincing of both CRC mucosa (TAF) and normal appearing mucosa, sampled at least 10 cm from the tumor [fibroblasts (FB)], and enzyme digestion as previously reported (31). Cell suspensions were seeded in culture wells and after 36 h non-adherent cells were removed. Adherent cells showing a fibroblast-like morphology were cultured for additional 7 day. At confluence, cell cultures were split and expanded as described (32). Phenotype for the indicated markers was analyzed at different time points and the expression of these markers was similar along the culture period of 2 months. Thus, MSC/TAF or MSC/FB expressed CD73, CD90, CD105, CD146, collagen I, vimentin (Figure S1 in Supplementary Material), bone sialoprotein, CD44, CD29, class I human leukocyte antigen (HLA-I), prolyl-4-hydroxylase, alkaline phoshatase, fibroblast activation protein, smooth muscle actin, the transcription marker SOX-2, and osteopontin but not CD45, CD31, CD33, CD34, CD3, CD2, CD16, CD14, ICAM2, ICAM3, CD80, CD86, CD83, and HLA-DR (31, 32). Nanog, OCT3-4 SSE-4, and TRA-1-60 stem cell markers were negative in both types of MSC (not shown).

### Cytotoxicity Assay

Cytolytic assay was performed as described previously (32, 33). Target cells were colon mucosa-derived MSC (TAF or FB) or the CRC cell lines Caco2, HCT15, and SW480. All these targets were cultured in RPMI 1640 24 h prior to the cytolytic assay and used in cytotoxicity experiments at the effector:target (E:T) ratio of 20:1. Reverse cytotoxicity was performed using the anti-NKG2D, the anti-DNAM1, the anti-CD16, the anti-NKp46, the anti-CD3, or the anti-CD56 mAbs, all at 2 µg/ml, and the FcγR positive murine P815 cell line [antibody-triggered cellular cytotoxicity (ATCC)], as described (33, 34) at 5:1 E:T ratio. Cells were labeled with ^51^Cr, washed, and seeded with effector cells. ^51^Cr-specific release was calculated as: experimental release (counts) − spontaneous release (counts)/maximum release (counts) − spontaneous release (counts). Maximum and spontaneous release were calculated as described (33, 34). In some experiments, saturating amounts of mAbs to NKG2D or DNAM1 or LFA1 and ICAM1 (2 µg/ml) were added. Target cells were also tested for their reactivity with the Fc chimeras (soluble receptors fused with the Fc of human immunoglobulins) NKG2D-Fc, DNAM1-Fc, NKp30-Fc, NKp44-Fc, and NKp46-Fc (R&D System), to determine the expression of ligands for these NK cell-activating receptors. NKp30-Fc, NKp44-Fc, and NKp46-Fc did not react with target cells (not shown). The expression of NKG2D-L and DNAM1-L on target cells was further confirmed with specific antibodies to MICA and ULBP3 (NKG2D-L) or CD115 (poliovirus receptor, PVR, as DNAM1-L) by immunofluorescence and FACS analysis (Figures S2 and S3 in Supplementary Material). In ADCC, the anti-EGFR cetuximab or the anti-CD20 rituximab humanized antibody (2 µg/ml) were added at the onset of the cytolytic assay, performed at the E:T ratio of 5:1. In another series of experiments, sorted NKp46^+^CD3^−^ cells were expanded in IL-2-containing medium and used either in ATCC as above or in cytotoxicity assay against HLA-I^+^ Caco2 or HLA-I^−^ HCT15 CRC cell line in a ^51^Cr release assay, or against autologous TAF: in this case, the crystal violet viability assay was used to evaluate target cell damage (35).

### Immunofluorescence and Cytofluorimetric Analysis

Immunofluorescence on either lymphocytes or colon MSC or CRC cell lines was performed with the indicated fluorophore-conjugated mAbs or primary mAbs followed by the addition of anti-isotype-specific goat anti-mouse (GAM) antisera (Southern Biotechnology, CA, USA) conjugated with AlexaFluor647 (Invitrogen, Thermo Fisher Scientific) as indicated. Control samples were stained with fluorophore-conjugated isotype control mAbs or isotype-matched irrelevant mAb (Becton Dickinson, Paolo Alto, CA, USA) followed by anti-isotype specific GAM-AlexaFluor647. To assess the reactivity of Fc chimeras, cetuximab or rituximab an anti-human immunoglobulin antiserum conjugated with AlexaFluor647 was used (Thermo Fisher Scientific). Samples were run on a CyAn ADP cytofluorimeter (Beckman-Coulter, Pasadena, CA, USA), analyzed with the Summit computer program (Beckman-Coulter), and are expressed as Log fluorescence intensity vs number of cells.

### Co-Cultures of Colon TAF, FB, and NK Cells

2 × 10^4^/sample MSC were co-cultured with NK cells at the NK:MSC ratio of 5:1 in 96W-microwell flat-bottomed plates (this was the first NK:MSC ratio at which an evident inhibition of NKG2D upregulation was detected; this ratio was determined in preliminary titration experiment starting from NK:MSC ratio of 20:1, 10:1, 5:1, 2:1, and 1:1) with 10 ng/ml IL-2 for 6 days and then analyzed for the expression and function of NKG2D and DNAM1 activating receptors by immunofluorescence and reverse cytotoxicity [or antibody-triggered cellular cytotoxicity (ATCC)]. In some experiments, NK cells were seeded on Millicell transwell (TW) with 0.3 µm pores (Millipore Corporation, Billerica, MA, USA) put into 24w plates with TAF or FB (6 × 10^4^/sample, the same number/area of culture well used for co-cultures of NK cells in direct contact with MSC) seeded in the lower chamber of the well, to avoid the contact with NK cells. Supernatants (SN) were collected from TAF cultures after 48 h for transforming growth factor (TGF)β measurement by ELISA (eBioscences, Thermo Fischer Scientific), after treatment for 10 min of each SN with 1N HCl as described (36). Results were normalized to a standard curve and expressed as picogram per milliliter.

### Immunohistochemistry

Paraffin-embedded samples from six CRC patients were analyzed. Immunohistochemistry was performed on 6-μm-thin sections, deparaffinized in xylene, and treated with Peroxo-Block (Thermo Fisher Scientific) to quench endogenous peroxidase, followed by Ultra V Block reagent (Ultravision Detection System, BioOptica, Thermo Fisher Scientific). Cetuximab and rituximab humanized antibodies were biotinylated using EZ-Link Sulfo-NHS-LC-Biotin (Thermo Fisher Scientific), according to the manufacturer’s instruction. These reagents were then added overnight to the slides, compared to the anti-vimentin mAb or to an isotypic unrelated antibody used as negative control (Dako Cytomation, Milan, Italy). Slides were then washed and incubated for 30 min with HRP-conjugated streptavidin (Thermo Fisher Scientific) and the reaction was developed using 3,3′-diaminobenzidine as chromogen. Subsequently, slides were counterstained with hematoxylin, cover-slipped with Eukitt (Bioptica, Thermo Fisher Scientific), and analyzed under an Leica DM MB2 microscope (Wetzlar, Germany) equipped with a charged coupled device camera (Olympus DP70, Tokyo, Japan) with a 20× or 40× objective.

### cDNA Reverse Transcription and Quantitative Real-Time PCR (Q-RT-PCR)

Natural killer cells cultured with IL-2 and colon MSC at the NK:MSC ratio of 5:1 were harvested at different time points (0, 3, 6, and 24 h) and analyzed for the expression of NKG2D receptor mRNA by Q-RT-PCR, as described (35). RNA was extracted with TriPure (Roche Diagnostic, Milan, Italy) and cDNA synthesis was performed with random primers. To verify quantitative Q-RT-PCR efficiency, decreasing amounts (50, 10, and 0.1 ng) of normal RNA were used for CT titration. Primers and probes for NKG2D were purchased by Applied Biosystem (Hs00183683_m1, Thermo Fisher Scientific). Q-RT-PCR was performed on the 7900HT FastRT-PCR system with the fluorescent Taqman method and normalized to 18s (Hs9999901_s1, Thermo Fisher Scientific). After subtracting the threshold cycle (C_T_) value for 18s from the C_T_ values of target genes, results were expressed as 2^−ΔΔCT^ expression ratio following MIQE guidelines.

### Statistical Analysis

Data were analyzed by applying the two-tailed Student’s *t* test at 99% confidence, or the non-parametric two-tailed *t* test (Mann–Whitney), using the GraphPad Prism software (La Jolla, CA, USA).

## Results

### TAF Isolated From CRC Inhibit NK Cell Antitumor Activity

We first analyzed the phenotype of MSC isolated from the tumor (TAF) and from tumor free mucosa (FB). We found that TAF and FB expressed CD73, CD90, CD105, and CD146, collagen I or vimentin and ICAM1 (Figures S1A,B in Supplementary Material, one representative case and Figures S1C,D in Supplementary Material, mean of six different cases). Moreover, TAF and FB reacted with the NKG2D and DNAM1 receptors when used as chimeric molecules and with antibodies to the NKG2D-L (MICA and ULBP3) and the DNAM1-L (PVR) (Figures S1B,D in Supplementary Material). Then, we analyzed whether co-culture of NK cells with CRC-derived MSC could affect the killing of established CRC cell lines.

Among a panel of 14 cell lines (see Materials and Methods), all reacting with Fc-DNAM1 chimera and all but T84 and Colo205 cell lines, with Fc-NKG2D chimera (Figure S2 in Supplementary Material) we chose the three representative CRC cell lines Caco2, HCT15, and SW480 expressing the adhesion molecule ICAM1 (Figure S2 in Supplementary Material) and the ligands of NKG2D (MICA and ULBPs) and DNAM1 activating receptors (PVR, Figure S3 in Supplementary Material). Moreover, Caco2 was selected as it does not display any mutation of KRAS, BRAF, and PI3K genes, while HCT15 and SW480 are mutated for KRAS (28). The Fc-NKp30, Fc-NKp44, and Fc-NKp46 chimeras did not react with the three analyzed CRC cell lines (not shown).

**Figure 1:**
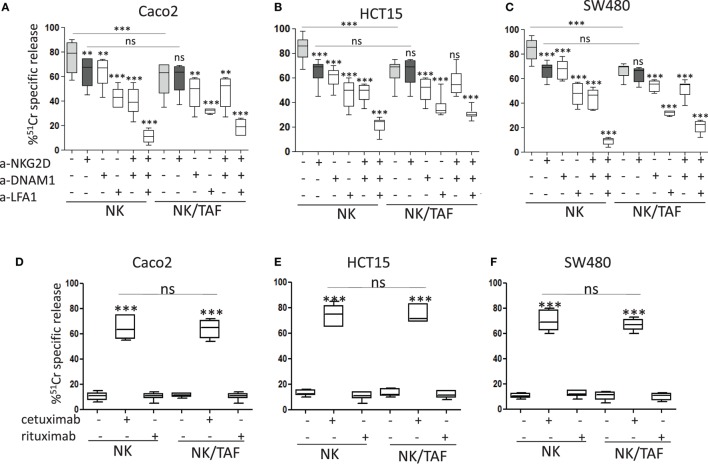
Natural killer (NK) cell killing of CRC lines is inhibited by tumor-associated fibroblasts (TAF). The carcinoma cell line Caco2 **(A)**, HCT15 **(B)**, and SW480 **(C)** were analyzed for their sensitivity to IL-2-activated NK cell mediated lysis [effector:target (E:T) ratio 20:1] without (light gray whiskers) or with monoclonal antibodies to NKG2D (dark gray whiskers) or DNAM1 or lymphocyte function associated (LFA)1 (2 µg/ml), alone or in combination. **(D–F)** NK cell activity (E:T ratio 5:1) against Caco2 **(D)**, HCT15 **(E)**, and SW480 **(F)** in the absence or in the presence of cetuximab or rituximab (2 µg/ml). NK cells were either cultured alone (NK) or derived from co-cultures with mesenchymal stromal cells from CRC mucosa (NK/TAF). Results are shown as percentage of ^51^Cr-specific release and they are the mean with boxes and whiskers min to max of six experiments with NK cells derived from six distinct co-cultures with TAF. In each panel: ***p < 0.0001; **p < 0.001; ns, not significant.

We found that IL-2-activated NK cells derived from co-cultures with tumor-derived MSC (NK/TAF) exerted lower cytotoxicity of Caco2, HCT15, and SW480 CRC cell lines, compared to NK cells cultured with IL-2 alone that were efficiently able to lyse CRC (Figures 1A–C, NK/TAF vs NK in each panel). NKG2D, DNAM1, and LFA1 molecules were all involved in CRC recognition and killing; indeed, the addition of mAbs against these molecules efficiently reduced target cell lysis and the simultaneous covering of all these receptors almost abolished NK cell mediated cytotoxicity (Figures 1A–C). Of note, the anti-NKG2D mAb did not inhibit tumor cell lysis exerted by NK/TAF cells (Figures 1A–C, dark vs light gray), suggesting that NKG2D-mediated recognition is impaired, at variance with anti-DNAM1 or anti-LFA1 mAbs (Figures 1A–C, white whiskers). These results indicate that CRC cells are recognized and killed by NK cells through the interaction of LFA1, NKG2D, and DNAM1 with their ligands expressed on target cells; further, TAF can affect NK cell mediated lysis of CRC cell lines, mainly interfering on the function of NKG2D. Noteworthily, NK cells harvested from NK/TAF co-cultures, could still be triggered by cetuximab, even at a very low E:T ratio (5:1), and exert ADCC against Caco2, HCT15, and SW480 cell lines (Figures 1D–F).

**Figure 2:**
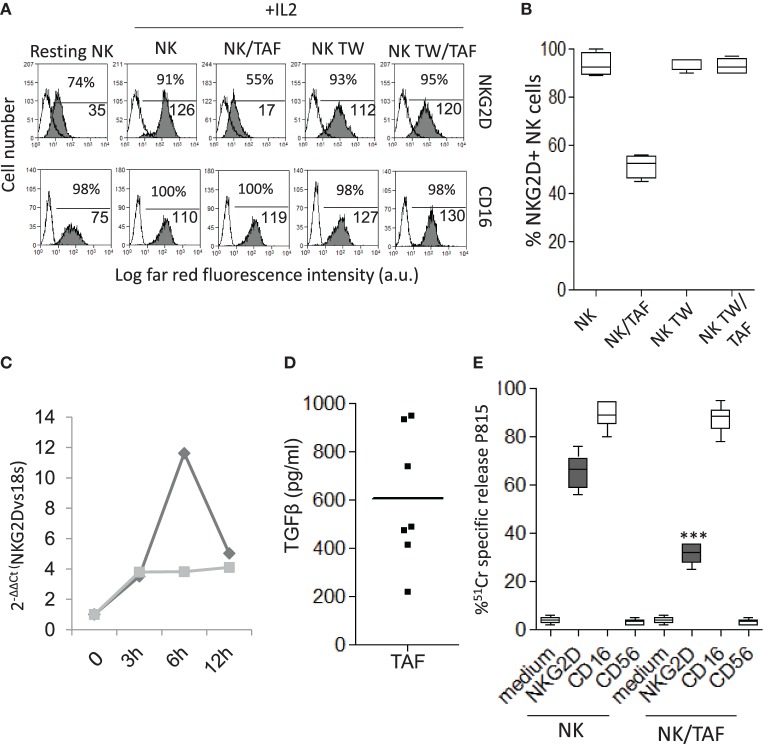
Tumor-associated fibroblasts (TAF) regulate the expression and function of NKG2D on activated natural killer (NK) cells. **(A)** NK cells were cultured with IL-2 for 6 days in contact with TAF (NK/TAF), or in the upper chamber of a transwell plate containing TAF in the lower chamber (NK TW/TAF), and analyzed for the expression of NKG2D (upper row, gray histogram) or CD16 (lower row, gray histogram) by immunofluorescence with the specific monoclonal antibodies (mAbs) followed by Alexafluor647 anti-isotype antiserum and FACS analysis. White histograms: cells labeled with the anti-isotype antiserum alone. NK alone (NK) or cultured in the transwell (NK TW) and freshly isolated (resting) NK were analyzed as controls. In each subpanel: percentage and the mean fluorescence intensity (MFI, a.u.) of positive cells. Results are expressed as Log far-red fluorescence intensity vs number of cells. **(B)** Percentage of NKG2D positive cells among NK cells alone (NK) or co-cultured with TAF (NK/TAF) in contact or separated by transwell (NK TW, NK TW/TAF). **(C)** IL-2-mediated upregulation of NKG2D mRNA was analyzed in NK cells alone (dark gray) or co-cultured with TAF (light gray), by quantitative real-time PCR at the indicated time points (0, 3, 6, and 12 h). Results are expressed as 2^−ΔΔCT^ and are representative of three independent experiments. **(D)** Transforming growth factor (TGF)β was measured by ELISA in the supernatant of TAF from six patients. Results are expressed as picogram per milliliter. **(E)** Reverse cytolysis (ATCC) of the target P815 cell line exerted by NK cells alone (NK) or co-cultured with TAF (NK/TAF), in the absence or presence of mAbs to the indicated NK cell surface molecules. Results are shown as percentage of ^51^Cr-specific release. **(B,E)** Mean with boxes and whiskers min to max of six experiments with NK cells derived from six distinct co-cultures with donor matched TAF. In each panel: ***p < 0.0001; ns, not significant.

It is of interest that the surface expression of NKG2D was markedly reduced in NK cells recovered from co-cultures with TAF (NK/TAF, Figure 2A, one representative experiment and Figure 2B, mean of six experiments with six different TAF); this decrease was not evident when NK and MSC were co-incubated for the same time but separated by a transwell system (NK TW/TAF, Figures 2A,B). No effect on CD16 (Figure 2A) or DNAM1 or LFA1 (not shown) expression was observed. Of note, co-incubation with TAF inhibited in NK cells the upregulation of mRNA coding for NKG2D triggered by IL-2 (Figure 2C); this finding suggests that the downregulation of NKG2D surface expression is mainly due to the impairment of the IL-2 mediated upregulation of mRNA for NKG2D and consequent reduced synthesis of NKG2D protein. More importantly, TGFβ, a cytokine involved in the downregulation of NKG2D protein expression (34) was detected in the SN of cultured TAF from six different patients (Figure 2D). In turn, specific inhibitors of prostaglandin (PG)E_2_ synthesis or indoleamine dioxigenase activity did not affect the TAF-mediated reduction of NKG2D expression (not shown). Finally, we observed that NK cells co-cultured with TAF showed a markedly lower cytolytic activity triggered by the NKG2D receptor in reverse killing (ATCC) against the P815 cell line, compared to that of NK cells cultured without TAF (Figure 2E). In this assay, cytolytic activity is due to the activation on the effector NK cell of a receptor recognized by a specific mAb that, in turn, binds to the Fc receptor of P815 cells acting as a bridge between effector and target. It is of note that triggering of NK cells through the CD16 receptor was very strong and similar in NK cells recovered from co-cultures with TAF to that displayed by NK cells alone (Figure 2E).

### CRC-Derived TAF Express EGFR and Can Be Targeted by the Anti-EGFR Humanized Antibody Cetuximab

**Figure 3:**
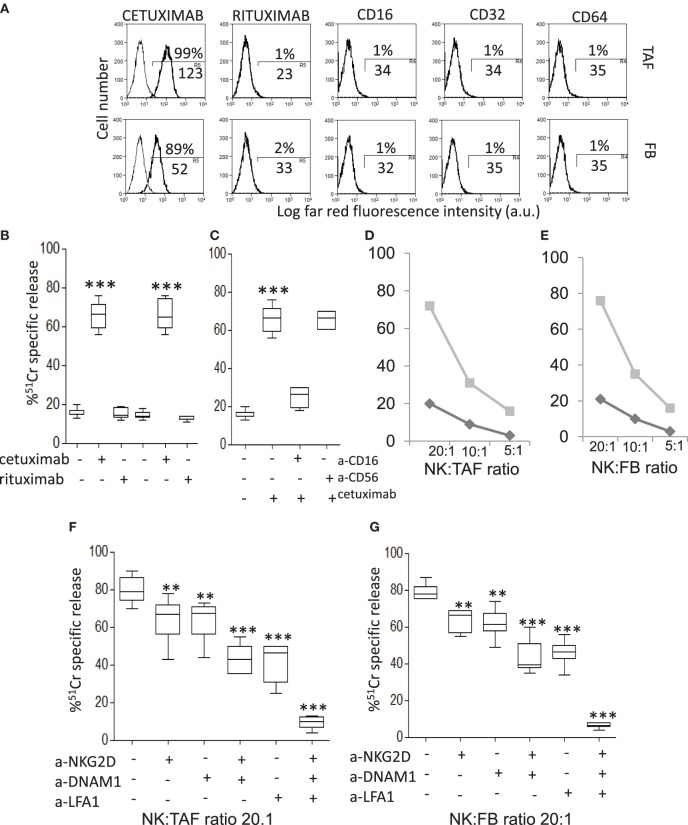
CRC-derived tumor-associated fibroblasts (TAF) can be targeted with cetuximab and killed by natural killer (NK) cells. **(A)** Mesenchymal stromal cells (MSC) from either CRC (TAF, upper row) or tumor free (FB, lower row) colon mucosa were assessed for the reactivity with cetuximab or rituximab (control isotype-matched antibody), as indicated, by indirect immunofluorescence with the specific Ab followed by AlexaFluor647 anti-human antiserum and FACS analysis. The expression on MSC of CD16 (FcγRIII), CD32 (FcγRII), and CD64 (FcγRI) antigens, upon immunofluorescence with specific mAbs followed by AlexaFluor647 goat anti-mouse (GAM), is also shown. In each panel: negative control (anti-human antiserum or GAM alone, thin black line) and reactivity with the indicated antibody (thick black line). Numbers in each subpanel are the percentage above the bar and the mean fluorescence intensity (MFI, below the bar, a.u.) of positive cells. **(B)** Antibody-dependent cellular cytotoxicity (ADCC) of TAF by NK cells at the NK:TAF ratio of 5:1 was evaluated with cetuximab or rituximab (2 µg/ml). **(C)** Cetuximab-mediated ADCC of TAF in the presence of the anti-CD16 monoclonal antibody (mAb) or the control anti-CD56 mAb (2 µg/ml). **(D,E)** Cytolysis exerted by resting (black) or IL-2-activated (gray) NK cells at the indicated effector:target ratio (20:1, 10:1, 5:1) against TAF **(D)** or FB **(E)**. **(F,G)** Effect of anti-NKG2D, anti-DNAM1, and anti-lymphocyte function associated (LFA)1 mAbs (2 µg/ml) on TAF **(F)** or FB **(G)** lysis exerted by IL-2-activated NK cells at 20:1 effector:target ratio. Results are shown as percentage of ^51^Cr-specific release and are the mean with boxes and whiskers min to max of six independent experiments with matched TAF and FB from six different patients. ***p < 0.0001; **p < 0.001.

**Figure 4:**
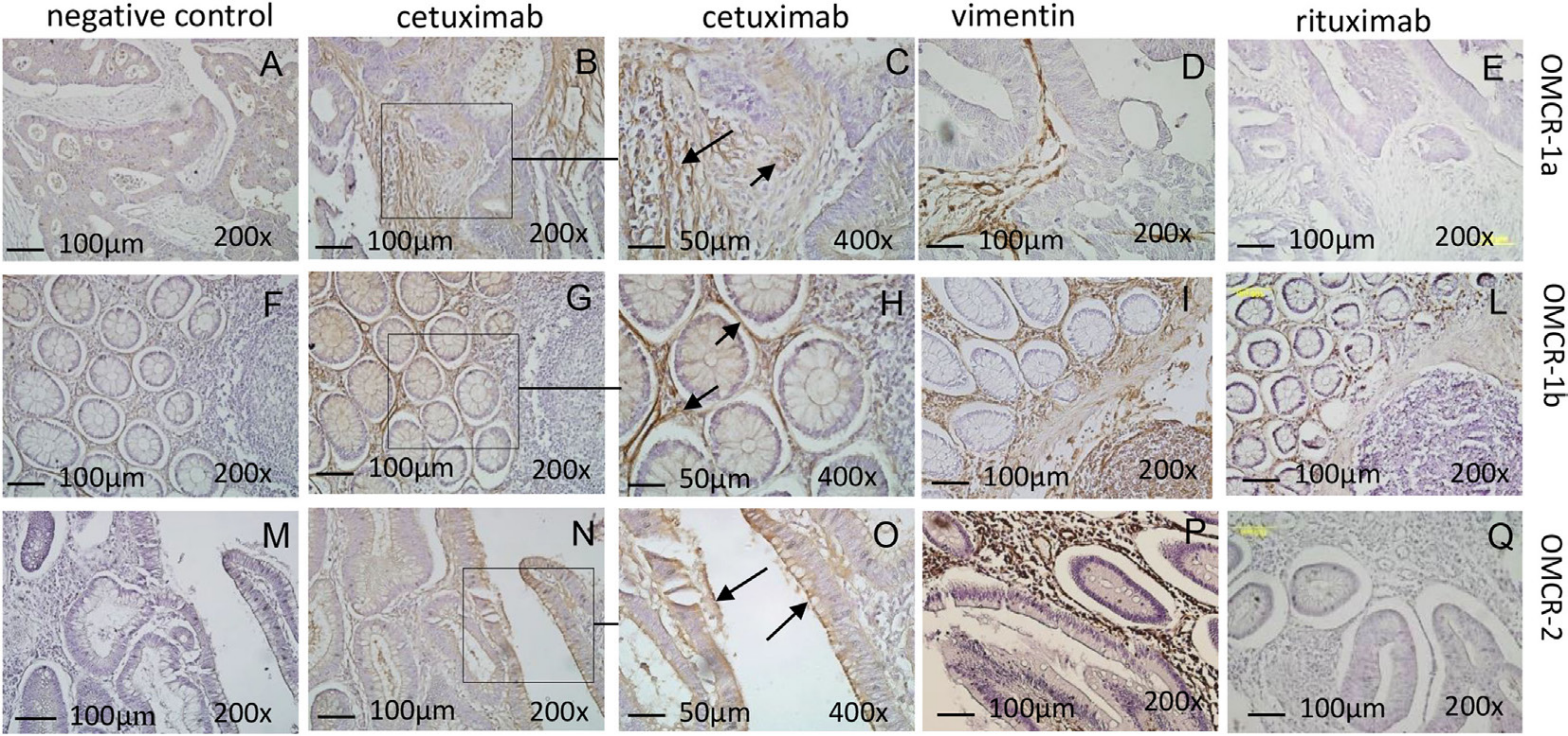
CRC tumor-associated fibroblasts express EGFR and are recognized by cetuximab at the tumor site. Reactivity of the humanized antibody cetuximab **(B,C,G,H,N,O)** was evaluated by immunohistochemistry on the CRC areas (OMCR-1a, upper row and OMCR-2, lower row) or the tumor free colon mucosa (OMCR-1b, middle row). Left panels **(A,F,M)**: negative control (anti-human antiserum alone). Upper and central rows: reactivity on mesenchymal stromal cells (MSC) [panels **(B,G)** and arrows in panel **(C,H)**]. Lower row: reactivity on epithelial cells [panel **(N)** and arrows in panel **(O)**]. **(C,H,O)** Enlargements of the squares indicated in panels **(B,G,N)**. MSC were identified by the specific expression of vimentin **(D,I,P)**. **(E,L,Q)** Reactivity of the control antibody rituximab. Data are representative of specimens from carcinoma or tumor free mucosa of six different patients. Images were taken with the Leica microscope DM MB2 microscope equipped with a charged coupled device camera (Olympus DP70); images were taken at 200× and 400× magnification.

As TAF/NK cross talk does not interfere with CD16-triggered NK cytotoxicity, we analyzed if the EGFR humanized antibody cetuximab can recognize MSC and lead to their killing. We have described that CRC-derived TAF primary cultures express EGFR (35): here, we confirmed that cetuximab can bind EGFR on these cells, either TAF or FB (Figure 3A shows one representative experiment out of six cases showing >90% positive cells with a MFI range of 65–130, not shown). Of note, we found that cetuximab reacted with TAF (identified by vimentin staining, Figures 4D,I,P) at the tumor site in CRC (Figure 4B, enlarged in Figure 4C) and stained also FB in non-neoplastic areas (Figure 4G, enlarged in Figure 4H). Epithelial cells expressed EGFR as well (Figures 4N,O). The specificity of cetuximab reaction with TAF in tumor specimens was also supported by the finding that the isotype-matched antibody rituximab (anti-CD20 humanized antibody) did not stain TAF or FB *in situ* (Figures 4E,L,Q) and that cultured TAF or FB did not express FCγRI, FCγRII, and FCγRIII (Figure 3A, CD64, CD32, and CD16). Staining with only anti-human immunoglobulin antiserum did not give any reactivity in CRC spacimens (Figures 4A,F,M).

Thus, we analyzed whether cetuximab can trigger the killing of CRC TAF or FB through the recognition of EGFR and the engagement of the CD16 receptor on NK cells. We found that cetuximab can induce peripheral blood freshly isolated NK cells to exert ADCC of TAF (Figure 3B). This effect was dependent on the engagement of CD16 on NK cells as the covering of this receptor with a specific anti-CD16 mAb, but not that of CD56, strongly inhibit cetuximab-mediated ADCC of TAF (Figure 3C). Similar results were obtained using FB (not shown). NK cells activated with IL-2 can also recognize and kill both TAF and FB, while freshly isolated NK cells exerted a very low cytotoxic effect (about 20%), in the absence of cetuximab, even at high E:T ratio (20:1, Figures 3D,E). The cytolytic function of activated NK cells was dependent on the interaction of LFA1, NKG2D, and DNAM1 with their ligands, as specific antibodies to these molecules can inhibit TAF (Figure 3F) or FB (Figure 3G) lysis and the simultaneous covering of all these three NK cell receptors can abolish cell lysis (Figures 3F,G). These findings suggest that TAF can be recognized and lysed by activated, but not resting NK cells that, in turn, can be triggered to kill TAF by cetuximab.

### CRC Lymphocyte Infiltrate Is Enriched in NKp46^+^ NK Cells: Interaction With Autologous TAF

**Figure 5:**
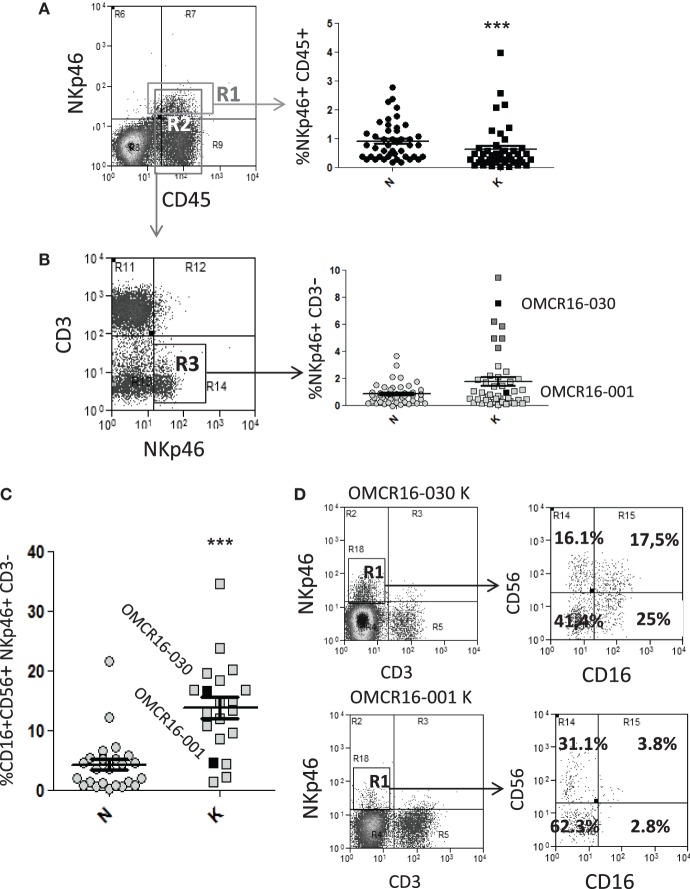
CRC cell suspensions contain infiltrating NKp46^+^ CD16^+^ natural killer (NK) cells. **(A,B)** CRC cell suspensions obtained from the tumor (K) or the non-tumor (N) areas of 45 specimens were stained with the anti-CD45 T205, the anti-CD3 JT3A, followed by FITC-anti IgM and APC-anti-IgG2a goat anti-mouse antiserum and PE-anti-NKp46 monoclonal antibodies (mAbs), and analyzed by FACS. Panel **(A)**, left: dot plot of immunofluorescence of a representative case of CRC. R1: gate on NKp46^+^CD45^+^cells, R2: gate on CD45^+^ cells. Panel **(A)**, right: percentage of NKp46^+^CD45^+^ cells in gate as R1 in 45 CRC. ***p < 0.001, non-parametric two-tailed *t* test Mann–Whitney. Panel **(B)**, left: dot plot of immunofluorescence on gate R2 of the sample depicted in panel **(A)**. R3: gate on NKp46^+^CD3^−^ cells. Panel **(B)**, right: percentage of NKp46^+^CD3^−^ cells in gate R3 in 45 CRC. The two cases indicated with black squares (OMCR16-030, above the mean and OMCR16-001, below the mean) are shown in more detail in panel **(D)**. Dark gray squares indicate the six cases, besides OMCR16-030, with NKp46^+^CD3^−^ cells between 4 and 10%, **(C)** CRC cell suspensions obtained from the tumor (K) or the non-tumor (N) areas of 20 specimens were stained with the PE/Dazzle-anti-CD3, PE-Cy7-anti-NKp46, PE-Cy5-anti-CD56, and Pacific Blue-anti-CD16 mAbs. CD56^+^CD16^+^ cells among the NKp46^+^CD3^−^ cell population. ***p < 0.001, non parametric two-tailed *t* test Mann–Whitney. Black squares: OMCR16-030 and OMCR16-001. **(D)** CD56 and CD16 expression (right dot plots) on the NKp46^+^CD3^−^ tumor cell population (K, gated in R1, left dot plots) of the two cases indicated with black squares in panels **(B,C)** (OMCR16-030 and OMCR16-001). In each quadrant: percentage of positive cells.

To verify the possible interactions between NK cell and MSC at the tumor site, we obtained NK cells or TAF from CRC cell suspensions and performed phenotypic analyses and co-culture experiments. In 45 CRC specimens, cell suspensions obtained from the tumor (K) or the non-tumor (N) areas, were cultured 12 h in flat-bottom plates to remove adherent cells; then, non-adherent cells were stained with anti-CD45 (leukocyte marker), anti-CD3 (T cell marker), and anti-NKp46 (NK cell marker) mAbs, followed by FACS analysis. As shown in Figure 5A, the mean percentage of NKp46^+^CD45^+^ cells was 0.93% in N or 0.64% in K (Figure 5A, right graph, gated on R1 in the left dot plot). To determine with more precision the NK cell infiltrate, NKp46^+^CD3^−^ cells were evaluated in the same K or N CRC samples, gated on CD45^+^ cells (gate R2 in Figure 5A): the mean percentage of NKp46^+^CD3^−^ cells was 1.8%, with seven cases between 4 and 10%, in the tumor areas (K), and 0.87% in N areas (in all but two cases between 2.1 and 3.8%) (Figure 5B, right graph, gated on R3 in the left dot plot). We further characterized the NK cell population, in 20 cases (out of 45, due to the shortage of cells) for the expression of CD16 and CD56 in NKp46^+^CD3^−^ cells with a four-color immune staining. Figure 5C shows a significantly higher percentage of CD16^+^CD56^+^ NKp46^+^CD3^−^ cells in the tumor (K) vs non-tumor (N) areas (mean value: 14.6 vs 4.9%). In Figure 5D, the two cases indicated with black squares in Figures 5B,C are shown, as examples of NK cell populations enriched or not in CD16^+^CD56^+^ (OMCR16-030 and OMCR16-001, respectively) cells in the tumor (K). In particular, OMCR16-030 specimen showed 42.5% (17.5% double positive and 25% single positive) CD16^+^ cells, while in OMCR16-001 sample CD16^+^ cells were 6.6% (3.8% double positive and 2.8% single positive).

**Figure 6:**
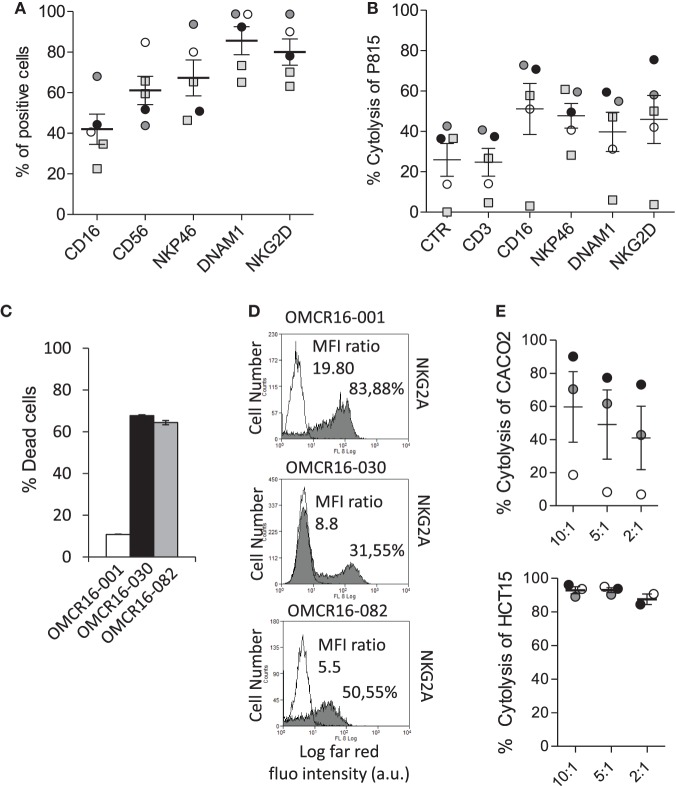
Sorted NKp46^+^ CD3^−^ cells from CRC give rise to natural killer (NK) cells with anti-tumor-associated fibroblasts (TAF) and antitumor activity. **(A)** Phenotype of cell suspensions from five CRC, by immunofluorescence with monoclonal antibodies (mAbs) against the indicated molecules (CD16, CD56, NKp46, DNAM1, and NKG2D) followed by anti-isotype APC-goat anti-mouse (GAM) antiserum and FACS analysis. Results are expressed as percentage of positive cells. Mean ± SEM of the five cases is shown. **(B)** Sorted NKp46^+^ CD3^−^ cells of 5 CRC, expanded with IL-2, were assayed in redirected killing (ATCC) against P815, in a ^51^Cr release assay, using mAbs recognizing CD16, NKp46, DNAM1, and NKG2D (CD3 was used as negative control). Results are expressed as percentage of P815 cell lysis. Mean ± SEM of the five cases is shown. **(C)** NKp46^+^ CD3^−^ cell populations from three CRC [OMCR16-030, OMCR16-082, and OMCR16-001, black, gray, and white, respectively, as the circles in panels **(A,B)**] were co-cultured for 24 h with autologous TAF at the ratio of 10:1 (NKp46^+^CD3^−^/TAF); then TAF viability was assessed by crystal violet assay and results expressed as percentage of dead cells. Mean ± SEM of triplicate samples is shown. **(D)** Expression of NKG2A by indirect immunofluorescence with the specific mAb (gray histograms), followed by isotype-specific APC-GAM and FACS analysis, on OMCR16-001, OMCR16-030, and OMCR16-082 NKp46^+^CD3^−^ cells. White histograms: negative control with APC-GAM alone. Results are expressed as log far-red fluorescence intensity (a.u.) vs number of cells. In each quadrant: percentage of positive cells and MFI ratio (MFI gray/MFI white histograms, a.u.). **(E)** Cytolytic activity of NKp46^+^CD3^−^ cell populations from OMCR16-030, OMCR16-082, and OMCR16-001 (black, gray, and white circles, respectively) against the HLA-I^+^ Caco2 (upper graph) or the HLA-I^−^ HCT15 (lower graph) CRC cell lines, in a ^51^Cr release assay. Results are expressed as percentage of specific cytolysis. Mean ± SEM of the three cases is shown.

Then, NKp46^+^CD3^−^ cells were sorted from five CRC (K), including OMCR16-030 and OMCR16-001, according to the protocol shown in Figure S4 in Supplementary Material. Sorted NKp46^+^CD3^−^ cells (98.5% purity) were expanded with IL-2 and used in ATCC against P815 (E:T ratio 2.5:1), in crystal violet assay with autologous TAF or in cytotoxicity assay (E:T ratio 10:1) against Caco2 or HCT15 CRC cell lines. These cell populations expressed CD16, NKG2D, DNAM1, and NKp46 (Figure 6A). As shown in Figure 6B, NKp46^+^CD3^−^ cells derived from four out of five CRC specimens could be elicited to activate ATCC *via* CD16, NKp46, DNAM1, and NKG2D (the anti-CD3 mAb used as negative control did not increase the spontaneous activity, identified as CTR in the figure), indicating that this cell population is potentially cytotoxic. Three cases (Figure 6, OMCR16-030, black circles and column; OMCR16-082, gray circles and column; and OMCR16-001, white circles and column) were studied in more detail as autologous TAF have been derived. OMCR16-030 and OMCR16-082 NKp46^+^CD3^−^ cells could kill most autologous TAF; by contrast, NKp46^+^CD3^−^ cells derived from OMCR16-001 population were not effective (Figure 6C). To explain this different behavior, we tested all the NKp46^+^CD3^−^ cell populations for the expression of some HLA-class I inhibitory receptors, such as KIR2D and NKG2A (5, 22, 37). We found a higher percentage of cells expressing the inhibitory molecule NKG2A among the NKp46^+^CD3^−^ population derived from OMCR16-001 CRC, compared to the same population sorted from OMCR16-030 and OMCR16-082 CRC (Figure 6D). Moreover, the MFI ratio vs the negative control (measure of the intensity of NKG2A expression) was more than twofold or threefold in OMCR16-001 NKp46^+^CD3^−^ cells. As NKG2A inhibitory signal is elicited by binding to HLA-I, we analyzed the ability of the different NKp46^+^CD3^−^ cell populations to lyse HLA-I^+^ (Caco2) or HLA-I^−^ (HCT15) CRC cell lines. As shown in Figure 6E, NKp46^+^CD3^−^ cells from OMCR16-001 case did not kill Caco2 (Figure 6E, upper graph, white circles) but was able to lyse HCT15 (Figure 6E, lower graph, white circles), at variance with OMCR16-030 and OMCR16-082 NKp46^+^CD3^−^ cells that efficiently killed both CRC cell lines (Figure 6E, upper graph vs lower graph, gray and black circles).

## Discussion

Here, we show that TAF from CRC reduce the NKG2D expression levels, through a direct cell-to-cell contact, and impair the NKG2D-mediated triggering of tumor cell lysis, sparing ADCC. Of note, the anti-EGFR therapeutic antibody cetuximab can overcome NK cell impairment, triggering the CD16-mediated ADCC of MSC.

In the tissue microenvironment, lymphocytes can be localized in contact with TAF, besides carcinoma cells, thus making possible that a negative signal impairs NK cell-mediated antitumor immune response *in vivo* (38–41). We found in the tumor a lymphoid infiltrate containing NKp46^+^CD3^−^ NK cells, according to other reports (42, 43). This population is enriched, in the tumor areas, in CD16^+^ cells, suggesting that ADCC can be elicited by humanized Ab-based therapy.

Along this line, NK cell isolated from the tumor and cultured with IL-2, could still be triggered *via* CD16, besides through the engagement of other NK cell activating receptors, including NKG2D. It is of note that sorted NKp46^+^CD3^−^ cells were able to kill autologous TAF; *in vivo*, this might represent a control mechanism to reduce TAF-mediated regulatory effect on NK cell function. In turn, the downregulation of NKG2D activation would be a mechanism to control NK cell-mediated recognition of MSC; this mechanism would limit the damage of NK cells to healthy FB. More importantly, the finding that resting peripheral blood NK cells can be triggered to kill MSC by cetuximab suggests an additional potential therapeutic effect of the humanized Ab to restore a full cytotoxic response against CRC (44–46). On the other hand, the effect of cetuximab administration in CRC patients might result in a lower therapeutic effect on the primary tumor due to the presence *in situ*, as we have shown by immunochemistry, of TAF that express EGFR and can subtract the antibody, thus reducing its availability (44–46). Cetuximab would be more efficacious in the metastatic tumor, where cancer cells are no longer protected by colon MSC. Nevertheless, we demonstrated that cetuximab is able to trigger NK cell-mediated ADCC, either against tumor cells or against TAF, thus potentially overcoming MSC-mediated immunosuppression.

We have also highlighted that both MSC and CRC cells are recognized by NK lymphocytes through the same receptors, namely LFA1, NKG2D, and DNAM1. This share of receptor molecules can have a role in favoring the escape of tumor cells from NK cell mediated killing. Indeed, in the tumor NK cells can bind to either CRC cells or MSC, instead of targeting primarily tumor cells and this can divert the antitumor immune response. At variance with our data, it has been shown that NCR, besides NKG2D and DNAM1 (47), are involved in the killing of CRC cells (48); however, in this report cancer initiating cells, but not the unselected tumor cell populations, were lysed through NCR. In addition, other authors described that reactivity with Fc-NCR becomes evident only when CRC cell lines are infected with parvovirus (49). Thus, our findings that the Fc-NCR chimeras do not react with the CRC cell lines analyzed, would suggest that different surface molecules are involved in the killing of cancer stem cells or virus-infected cells, compared to the large majority of tumor cells.

We noted that MSC from both carcinoma (TAF) and tumor free colon mucosa (FB) showed a similar behavior suggesting that the ability of inhibiting lymphocyte-mediated activities is an intrinsic and basic feature of MSC. A limitation to this interpretation of our results is represented by the fact that, to achieve a stock of cells for functional experiments, MSC were seeded *in vitro* and expanded in culture (4, 29, 31), leading to the loss of features, characterizing TAF or FB, present *in situ*. It has been reported that TAF from melanoma lesions can inhibit NK cell activation better than skin FB isolated from healthy donors (20); this inhibiting effect is reported to depend on the production of PGE_2_ during NK-TAF interaction. Our results indicate that TAF from CRC, matched with FB from the tumor free mucosa of the same patient, do not show different phenotypic or functional characteristics. However, in our study also FB come from a tissue potentially subjected to inflammatory factors released in the tumor and in the surrounding microenvironment, so that they might be considered not entirely healthy cells. These discrepancies could also be related to the use, in the cited report, of unmatched skin FB. Indeed, NK cells are sensitive to the inhibitory signal delivered by HLA-I (50, 51). Thus, the effect of FB from different donors on the same NK cell population, and *vice versa*, may depend on the fact that this population bear inhibiting HLA-I receptors at the cell surface that recognize different HLA-I alleles on FB. In support to this, we found that tumor-derived NK cells expressing high levels of the inhibitory receptor NKG2A did not aggress autologous TAF nor HLA-I^+^ CRC cells. In addition, we did not identify PGE_2_ as one of the factors responsible for inhibiting signals delivered to NK cells; rather, TGFβ released by MSC seems to be the factor potentially implied. This would suggest that the molecular mechanism underlying stromal-mediated inhibition is not the same for different tissue districts. According to our data, it has been reported that TGFβ can impair the IL-2-mediated upregulation of NKG2D mRNA (52–54). Furthermore, as demonstrated by experiments with millicell transwell chambers, the downregulation of NKG2D on NK cells was not detected when NK cells and MSC were not in direct contact and only soluble molecules present in co-culture supernatant could bind to NK cells. Thus, the reduced NKG2D surface expression and function is likely due to the effect of TGFβ rather than to soluble ligands which cover NKG2D molecule, affecting antibody reactivity (55).

In conclusion, our findings support that MSC may be a target for immunotherapies designed to favor antitumor lymphocyte effector functions (9); the use of cetuximab in anti-CRC therapeutic schemes should be useful to overcome MSC-mediated immunosuppression.

## Ethics Statement

This study was approved by the Institutional and regional ethical committee, PR163REG2014 and by the Regional Ethic Committee, CONSAZH780148/17/07/2015.

## Author Contributions

DC and RV performed cell isolation and culture, cytotoxicity and immunofluorescence assays; RB derived some MSC cell lines and discussed results; SC performed PCR; FL performed cell sorting; SM performed IHC; ER, SS, FG, and LM provided CRC specimens and histologic characterization; BR provided humanized antibodies; MZ and AP design the work, perform some experiments with MSC, and wrote the paper; and AP takes primary responsibility for the paper content.

## Conflict of Interest Statement

The authors declare that the research was conducted in the absence of any commercial or financial relationships that could be construed as a potential conflict of interest.

## Supplementary Material

Figure S1 Phenotype of cultured MSC isolated from colon carcinoma [tumor-associated fibroblasts (TAF)] and healthy mucosa (FB). MSC were isolated from colon carcinoma mucosa (TAF) and from tumor free mucosa (FB) of the same patient, cultured and analyzed for the expression of the indicated surface (CD73, CD90, CD105, and CD146) and cytoplasmic (collagen and vimentin) markers **(A,C)** or the NKG2D ligands or DNAM1 ligands [MICA, ULBP3, and poliovirus receptor (PVR)], intercellular adhesion molecule 1, or the Fc-NKG2D and the Fc-DNAM1 chimeras **(B,D)** by indirect immunofluorescence and FACS analysis. **(A,B)** One representative experiment: TAF or FB were stained with the monoclonal antibodies to the indicated molecules followed by AlexaFluor647-conjugated anti-isotype specific goat anti-mouse (gray histograms) or with the second reagent alone (negative control, white histogram). In each subpanel, the percentage of positive cells, above the bar set on the negative control, and the mean fluorescence intensity (MFI, below the bar) are indicated. Results are shown as Log far-red fluorescence intensity (arbitrary units, a.u.) vs cell number. **(C,D)** Results are expressed as percentage of positive cells and are the mean with boxes and whiskers min to max of six independent experiments with matched TAF (white boxes) and FB (gray boxes) from six different patients.

Figure S2 Expression of intercellular adhesion molecule (ICAM)1, NKG2D ligands (NKG2D-L) or DNAM1 ligands (DNAM1-L) on CRC cell lines. The carcinoma cell lines Caco2, HT29, HCT15, SW480, DLD1, HCT116, LS180 **(A)**, WiDr, LoVo, Colo205, Colo320 DMF, SW620, T84, and SW480 **(B)** were analyzed for the expression of ICAM1, with the specific monoclonal antibodies, or NKG2D-L or DNAM1-L with the Fc-NKG2D or Fc-DNAM1 chimeric molecules by immunofluorescence assay and FACS analysis. In each panel, the negative control (AlexaFluor647 goat anti-mouse for ICAM1 and AlexaFluor647 human antiserum for the chimeras, black histograms) vs positive samples (gray histograms) is shown. Data are expressed as Log far-red fluorescence intensity (arbitrary units, a.u.) vs number of cells.

Figure S3 Expression of MICA, ULBPs, or poliovirus receptor (PVR) on selected CRC cell lines. The carcinoma cell lines Caco2, HCT15, and SW480 were analyzed for the expression of MICA, ULBP1, ULBP2, ULBP3, and PVR with specific monoclonal antibodies by immunofluorescence assay and FACS analysis. In each panel, the negative control (AlexaFluor647 goat anti-mouse, white histograms) vs positive samples (gray histograms) is shown. Data are expressed as Log far-red fluorescence intensity (arbitrary units, a.u.) vs number of cells.

Figure S4 Sorting strategy for NKp46^+^CD3^−^ cells from CRC. NKP46^+^CD3^−^ cell sorting from the OMCR16-030 CRC is shown as an example. Representative gating strategy: plots show first the recognition of the population of interest, without doublets, than the target of sorting NKp46^+^cells on CD3^−^. **(A)** Gray dots are doublet 1 and 2 events [depicted in panels **(B,C)**] excluded on the basis of physical parameters; **(D)** dark gray dots are cells excluded on the basis of CD3 expression. **(E)** Gray dots are sorted NKp46^+^CD3^−^ cells. **(F)** CD16 and NKp46 expression (NKP46 PE-Cy7 vs CD16 Pacific Blue) on CD3^−^ cells sorted in panel **(E)**.

## References

[1] Di Nicola M Carlo-Stella C Magni M Milanesi M Stucchi C Cleris L. Human bone marrow stromal cells suppress T-lymphocyte proliferation induced by cellular or nonspecific mitogenic stimuli. Blood (2002) 99:3838–43. 10.1182/blood.V99.10.3838 11986244

[2] Aggarwal S Pittenger MF. Human mesenchymal stem cells modulate allogeneic immune cell responses. Blood (2005) 105:1815–22. 10.1182/blood-2004-04-1559 15494428

[3] Beyth S Borovsky Z Mevorach D Liebergall M Gazit Z Aslan H Human mesenchymal stem cells alter antigen-presenting cell maturation and induce T cell unresponsiveness. Blood (2005) 105:2214–9. 10.1182/blood-2004-07-2921 15514012

[4] Poggi A Prevosto C Massaro AM Negrini S Urbani S Pierri I Interaction between human natural killer cells and bone marrow stromal cells induces NK cell triggering. Role of NKp30 and NKG2D receptors. J Immunol (2005) 175:6352–60. 10.4049/jimmunol.175.10.6352 16272287

[5] Spaggiari GM Capobianco A Becchetti S Mingari MC Moretta L. Mesenchymal stem cell-natural killer cell interactions: evidence that activated NK cells are capable of killing MSCs, whereas MSCs can inhibit IL-2-induced NK-cell proliferation. Blood (2006) 107:1484–90. 10.1182/blood-2005-07-2775 16239427

[6] Sotiropoulou PA Perez SA Gritzapis AD Baxevanis CN Papamichail M. Interactions between human mesenchymal stem cells and natural killer cells. Stem Cells (2006) 24:74–85. 10.1634/stemcells.2004-0359 16099998

[7] Meisel R Zibert A Laryea M Gobel U Daubener U Dilloo D. Human bone marrow stromal cells inhibit allogeneic T-cell responses by indoleamine 2,3-dioxygenase-mediated tryptophan degradation. Blood (2004) 103:4619–21. 10.1182/blood-2003-11-3909 15001472

[8] Krampera M Mesenchymal stromal cell “licensing”: a multistep process. Leukemia (2011) 25:1408–14. 10.1038/leu.2011.108 21617697

[9] Poggi A Musso A Dapino I Zocchi MR. Mechanisms of tumor escape from immune system: role of mesenchymal stromal cells. Immunol Lett (2014) 159:55–72. 10.1016/j.imlet.2014.03.001 24657523

[10] Turley SJ Cremasco V Astarita JL. Immunological hallmarks of stromal cells in the tumour microenvironment. Nat Rev Immunol (2015) 15:669–82. 10.1038/nri3902 26471778

[11] Barnas JL Simpson-Abelson MR Brooks SP Kelleher RJ Bankert RB. Reciprocal functional modulation of the activation of T lymphocytes and fibroblasts derived from human solid tumors. J Immunol (2010) 185:2681–92. 10.4049/jimmunol.1000896 20686130

[12] Jackson KW Christiansen VJ Yadav VR Silasi-Mansat R Lupu F Awasthi V Suppression of tumor growth in mice by rationally designed pseudopeptide inhibitors of fibroblast activation protein and prolyloligopeptidase. Neoplasia (2015) 17:43–54. 10.1016/j.neo.2014.11.002 25622898 PMC4309729

[13] Nakayama H Miyazaki E Enzan H. Differential expression of high molecular weight caldesmon in colorectal pericryptal fibroblasts and tumourstroma. J Clin Pathol (1999) 52:785–6. 10.1136/jcp.52.10.785 10674042 PMC501579

[14] O’Malley G Heijltjes M Houston AM Rani S Ritter T Egan LJ Mesenchymal stromal cells (MSCs) and colorectal cancer – a troublesome twosome for the anti-tumour immune response? Oncotarget (2016) 7:60752–74. 10.18632/oncotarget.11354 27542276 PMC5312417

[15] Hogan NM Dwyer RM Joyce MR Kerin MJ. Mesenchymal stem cells in the colorectal tumor microenvironment: recent progress and implications. Int J Cancer (2012) 131:1–7. 10.1002/ijc.27458 22290082

[16] Li X Wang Y Zhao Y Yang H Tong A Zhao C Immunotherapy of tumor with vaccine based on basic fibroblast growth factor-activated fibroblasts. J Cancer Res Clin Oncol (2014) 140:271–80. 10.1007/s00432-013-1547-5 24322179 PMC11823931

[17] Lee J Fassnacht M Nair S Boczkowski D Gilboa E. Tumor immunotherapy targeting fibroblast activation protein, a product expressed in tumor-associated fibroblasts. Cancer Res (2005) 65:11156–63. 10.1158/0008-5472.CAN-05-2805 16322266

[18] Loeffler M Krüger JA Niethammer AG Reisfeld RA Targeting tumor associated fibroblasts improves cancer chemotherapy by increasing intratumoral drug uptake. J Clin Invest (2006) 116:1955–62. 10.1172/JCI26532 16794736 PMC1481657

[19] Chen M Xiang R Wen Y Xu G Wang C Luo S A whole-cell tumor vaccine modified to express fibroblast activation protein induces antitumor immunity against both tumor cells and cancer-associated fibroblasts. Sci Rep (2015) 5:14421. 10.1038/srep14421 26394925 PMC4585784

[20] Balsamo M Scordamaglia F Pietra G Manzini C Cantoni C Boitano M Melanoma-associated fibroblasts modulate NK cell phenotype and antitumor cytotoxicity. Proc Natl Acad Sci U S A (2009) 106:20847–52. 10.1073/pnas.0906481106 19934056 PMC2791633

[21] Takahashi H Sakakura K Kawabata-Iwakawa R Rokudai S Toyoda M Nishiyama M Immunosuppressive activity of cancer-associated fibroblasts in head and neck squamous cell carcinoma. Cancer Immunol Immunother (2015) 64:1407–17. 10.1007/s00262-015-1742-0 26201938 PMC11029788

[22] Bryceson YT March ME Ljunggren HG Long EO. Activation, coactivation, and costimulation of resting human natural killer cells. Immunol Rev (2006) 214:73–91. 10.1111/j.1600-065X.2006.00457.x 17100877 PMC3845883

[23] Nausch N Cerwenka A. NKG2D ligands in tumor immunity. Oncogene (2008) 27:5944–58. 10.1038/onc.2008.272 18836475

[24] Martinet L Smyth MJ. Balancing natural killer cell activation through paired receptors. Nat Rev Immunol (2015) 15:243–54. 10.1038/nri3799 25743219

[25] Shen Y Lu C Tian W Wang L Cui B Jiao Y Possible association of decreased NKG2D expression levels and suppression of the activity of natural killer cells in patients with colorectal cancer. Int J Oncol (2012) 40:1285–90. 10.3892/ijo.2011.1315 22200673 PMC3584522

[26] Tallerico R Garofalo C Carbone E. A new biological feature of natural killer cells: the recognition of solid tumor-derived cancer stem cells. Front Immunol (2016) 7:179. 10.3389/fimmu.2016.00179 27242786 PMC4861715

[27] Veluchamy JP Spanholtz J Tordoir M Thijssen VL Heideman DA Verheul HM Combination of NK cells and cetuximab to enhance anti-tumor responses in RAS mutant metastatic colorectal cancer. PLoS One (2016) 11:e0157830. 10.1371/journal.pone.0157830 27314237 PMC4912059

[28] Mouradov D Sloggett C Jorissen RN Love CG Li S Burgess AW Colorectal cancer cell lines are representative models of the main molecular subtypes of primary cancer. Cancer Res (2014) 74:3238–47. 10.1158/0008-5472.CAN-14-0013 24755471

[29] Prevosto C Zancolli M Canevali P Zocchi MR Poggi A. Generation of CD4^+^ or CD8^+^ regulatory T cells upon mesenchymal stem cell-lymphocyte interaction. Haematologica (2007) 92:881–8. 10.3324/haematol.11240 17606437

[30] Zocchi MR Costa D Venè R Tosetti F Ferrari N Minghelli S Zoledronate can induce colorectal cancer microenvironment expressing BTN3A1 to stimulate effector γδ T cells with anti-tumor activity. Oncoimmunology (2017) 6(3):e1278099. 10.1080/2162402X.2016.1278099 28405500 PMC5384426

[31] Benelli R Venè R Minghelli S Carlone S Gatteschi B Ferrari N. Celecoxib induces proliferation and Amphiregulin production in colon subepithelial myofibroblasts, activating erk1-2 signaling in synergy with EGFR. Cancer Lett (2013) 328:73–82. 10.1016/j.canlet.2012.09.008 23010081

[32] Musso A Zocchi MR Poggi A. Relevance of the mevalonate biosynthetic pathway in the regulation of bone marrow mesenchymal stromal cell-mediated effects on T-cell proliferation and B-cell survival. Haematologica (2011) 96:16–23. 10.3324/haematol.2010.031633 20884711 PMC3012760

[33] Spaggiari GM Contini P Carosio R Arvigo M Ghio M Oddone D Soluble HLA class I molecules induce natural killer cell apoptosis through the engagement of CD8: evidence for a negative regulation exerted by members of the inhibitory receptor superfamily. Blood (2002) 99:1706–14. 10.1182/blood.V99.5.1706 11861287

[34] Zocchi MR Camodeca C Nuti E Rossello A Venè R Tosetti F ADAM10 new selective inhibitors reduce NKG2D ligand release sensitizing Hodgkin lymphoma cells to NKG2D-mediated killing. Oncoimmunology (2015) 5:e1123367. 10.1080/2162402X.2015.1123367 27467923 PMC4910733

[35] Venè R Tosetti F Minghelli S Poggi A Ferrari N Benelli R. Celecoxib increases EGF signaling in colon tumor associated fibroblasts, modulating EGFR expression and degradation. Oncotarget (2015) 6:12310–25. 10.18632/oncotarget.3678 25987127 PMC4494940

[36] Zocchi MR Catellani S Canevali P Tavella S Garuti A Villaggio B High ERp5/ADAM10 expression in lymph node microenvironment and impaired NKG2D ligands recognition in Hodgkin lymphomas. Blood (2012) 119:1479–89. 10.1182/blood-2011-07-370841 22167753

[37] Navarro F Llano M Bellón T Colonna M Geraghty DE López-Botet M. The ILT2(LIR1) and CD94/NKG2A NK cell receptors respectively recognize HLA-G1 and HLA-E molecules co-expressed on target cells. Eur J Immunol (1999) 29(1):277–83. 10.1002/(SICI)1521-4141(199901)29:01<277::AID-IMMU277>3.0.CO;2-4 9933109

[38] Cirri P Chiarugi P. Cancer-associated-fibroblasts and tumour cells: a diabolic liaison driving cancer progression. Cancer Metastasis Rev (2012) 31:195–208. 10.1007/s10555-011-9340-x 22101652

[39] Quante M Tu SP Tomita H Gonda T Wang SS Takashi S Bone marrow-derived myofibroblasts contribute to the mesenchymal stem cell niche and promote tumor growth. Cancer Cell (2011) 19:257–72. 10.1016/j.ccr.2011.01.020 21316604 PMC3060401

[40] Raffaghello L Vacca A Pistoia V Ribatti D. Cancer associated fibroblasts in hematological malignancies. Oncotarget (2015) 6:2589–603. 10.18632/oncotarget.2661 25474039 PMC4413603

[41] Lazennec G Jorgensen C. Concise review: adult multipotent stromal cells and cancer: risk or benefit? Stem Cells (2008) 26:1387–94. 10.1634/stemcells.2007-1006 18388305 PMC2572832

[42] Halama N Braun M Kahlert C Spille A Quack C Rahbari N Natural killer cells are scarce in colorectal carcinoma tissue despite high levels of chemokines and cytokines. Clin Cancer Res (2011) 17(4):678–89. 10.1158/1078-0432.CCR-10-2173 21325295

[43] Tomasello E Yessaad N Gregoire E Hudspeth K Luci C Mavilio D Mapping of NKp46(+) cells in healthy human lymphoid and non-lymphoid tissues. Front Immunol (2012) 3:344. 10.3389/fimmu.2012.00344 23181063 PMC3501723

[44] Nakadate Y Kodera Y Kitamura Y Shirasawa S Tachibana T Tamura T KRAS mutation confers resistance to antibody-dependent cellular cytotoxicity of cetuximab against human colorectal cancer cells. Int J Cancer (2014) 134:2146–55. 10.1002/ijc.28550 24136682

[45] Vanneman M Dranoff G. Combining immunotherapy and targeted therapies in cancer treatment. Nat Rev Cancer (2012) 12:237–51. 10.1038/nrc3237 22437869 PMC3967236

[46] Scott AM Wolchok JD Old LJ. Antibody therapy of cancer. Nat Rev Cancer (2012) 12:278–87. 10.1038/nrc3236 22437872

[47] Zhang Z Su T He L Wang H Ji G Liu X Identification and functional analysis of ligands for natural killer cell activating receptors in colon carcinoma. Tohoku J Exp Med (2012) 226(1):59–68. 10.6120/tjem.226.59 22189020

[48] Tallerico R Todaro M Di Franco S Maccalli C Garofalo C Sottile R Human NK cells selective targeting of colon cancer-initiating cells: a role for natural cytotoxicity receptors and MHC class I molecules. J Immunol (2013) 190(5):2381–90. 10.4049/jimmunol.1201542 23345327

[49] Bhat R Rommelaere J. NK-cell-dependent killing of colon carcinoma cells is mediated by natural cytotoxicity receptors (NCRs) and stimulated by parvovirus infection of target cells. BMC Cancer (2013) 13:367. 10.1186/1471-2407-13-367 23902851 PMC3733944

[50] Manser AR Weinhold S Uhrberg M. Human KIR repertoires: shaped by genetic diversity and evolution. Immunol Rev (2015) 267:178–96. 10.1111/imr.12316 26284478

[51] Robinette ML Colonna M. Innate lymphoid cells and the MHC. HLA (2016) 87:5–11. 10.1111/tan.12723 26812060 PMC5658205

[52] Lee JC Lee KM Kim DW Heo DS. Elevated TGF-beta1 secretion and down-modulation of NKG2D underlies impaired NK cytotoxicity in cancer patients. J Immunol (2004) 172:7335–40. 10.4049/jimmunol.172.12.7335 15187109

[53] Sun C Fu B Gao Y Liao X Sun R Tian Z TGF-β1 down regulation of NKG2D/DAP10 and 2B4/SAP expression on human NK cells contributes to HBV persistence. PLoS Pathog (2012) 8(3):e1002594 10.1371/journal.ppat.1002594 22438812 PMC3305436

[54] Castriconi R Cantoni C Della Chiesa M Vitale M Marcenaro E Conte R Transforming growth factor beta 1 inhibits expression of NKp30 and NKG2D receptors: consequences for the NK-mediated killing of dendritic cells. Proc Natl Acad Sci U S A (2003) 100:4120–5. 10.1073/pnas.0730640100 12646700 PMC153058

[55] Klöss S Chambron N Gardlowski T Weil S Koch J Esser R Cetuximab reconstitutes pro-inflammatory cytokine secretions and tumor-infiltrating capabilities of sMICA-inhibited NK cells in HNSCC tumor spheroids. Front Immunol (2015) 6:543. 10.3389/fimmu.2015.00543 26579120 PMC4629470

