# Supplementary figures and images for: Targeting the Epidermal Growth Factor Receptor Can Counteract the Inhibition of Natural Killer Cell Function Exerted by Colorectal Tumor-Associated Fibroblasts
Source: Front Immunol. 2018 May 29;9:1150. doi: 10.3389/fimmu.2018.01150 (PMC5992415; doi:10.3389/fimmu.2018.01150)

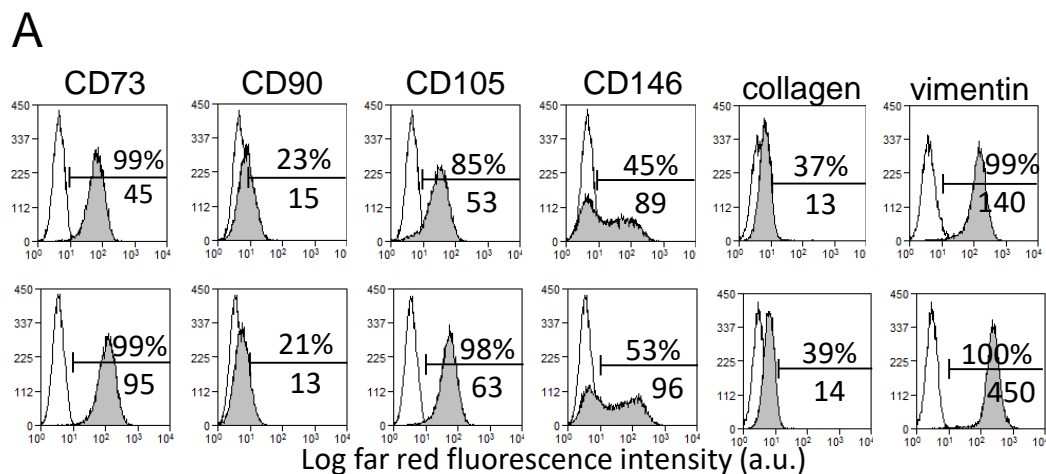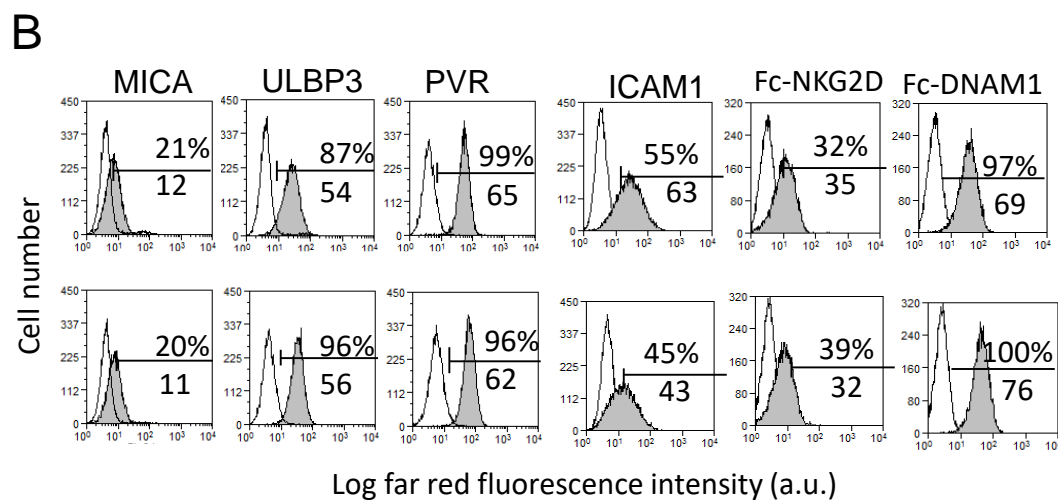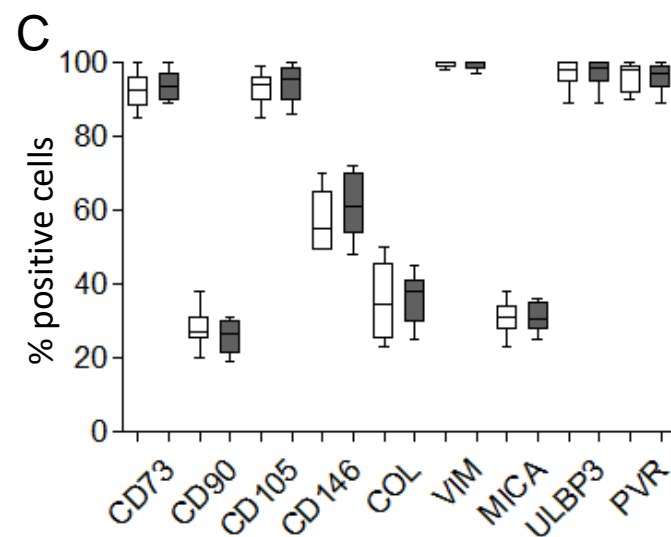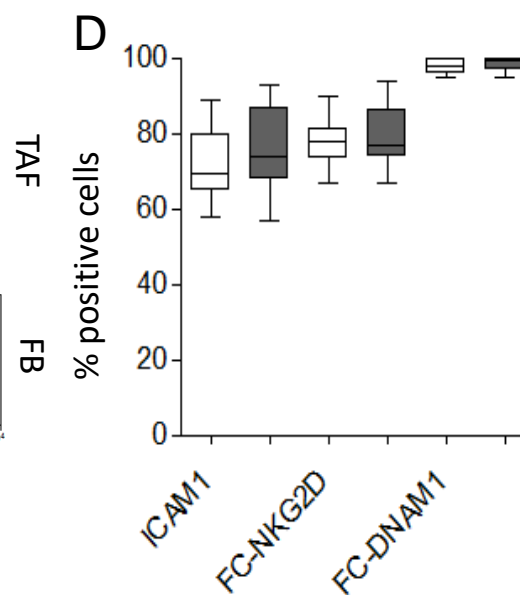

Supplement: Figure S1 — Phenotype of cultured MSC isolated from colon carcinoma [tumor-associated fibroblasts (TAF)] and healthy mucosa (FB). MSC were isolated from colon carcinoma mucosa (TAF) and from tumor free mucosa (FB) of the same patient, cultured and analyzed for the expression of the indicated surface (CD73, CD90, CD105, and CD146) and cytoplasmic (collagen and vimentin) markers (A,C) or the NKG2D ligands or DNAM1 ligands [MICA, ULBP3, and poliovirus receptor (PVR)], intercellular adhesion molecule 1, or the Fc-NKG2D and the Fc-DNAM1 chimeras (B,D) by indirect immunofluorescence and FACS analysis. (A,B) One representative experiment: TAF or FB were stained with the monoclonal antibodies to the indicated molecules followed by AlexaFluor647-conjugated anti-isotype specific goat anti-mouse (gray histograms) or with the second reagent alone (negative control, white histogram). In each subpanel, the percentage of positive cells, above the bar set on the negative control, and the mean fluorescence intensity (MFI, below the bar) are indicated. Results are shown as Log far-red fluorescence intensity (arbitrary units, a.u.) vs cell number. (C,D) Results are expressed as percentage of positive cells and are the mean with boxes and whiskers min to max of six independent experiments with matched TAF (white boxes) and FB (gray boxes) from six different patients. [file Image_1.PDF]

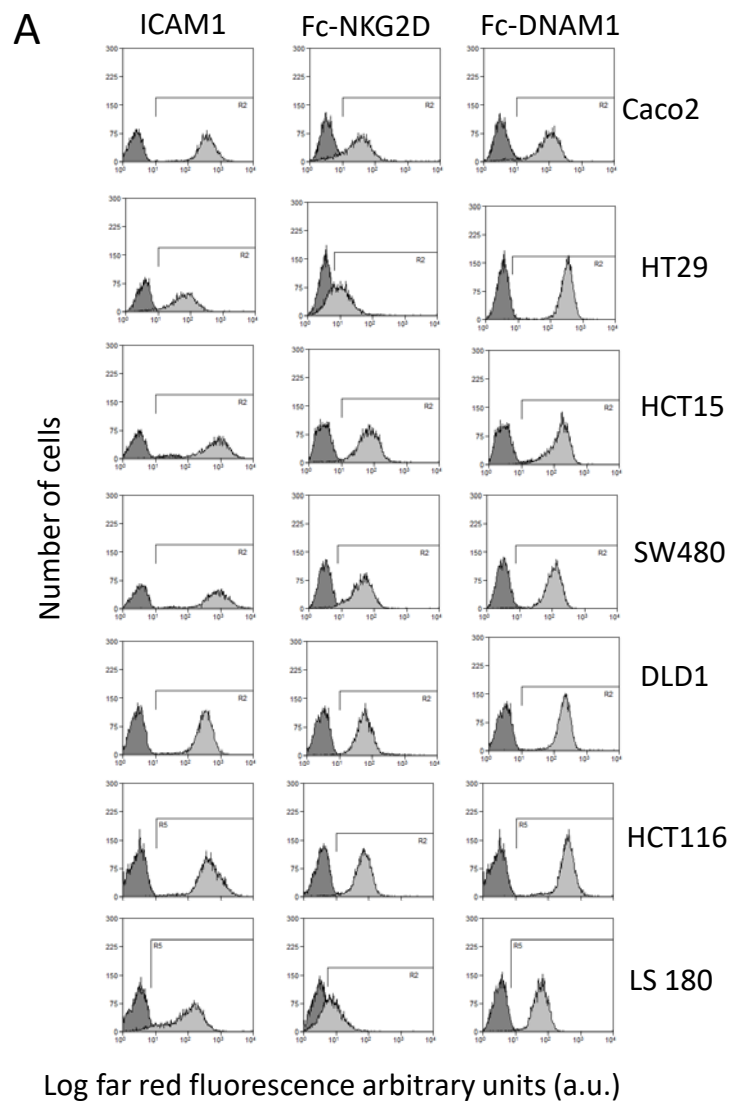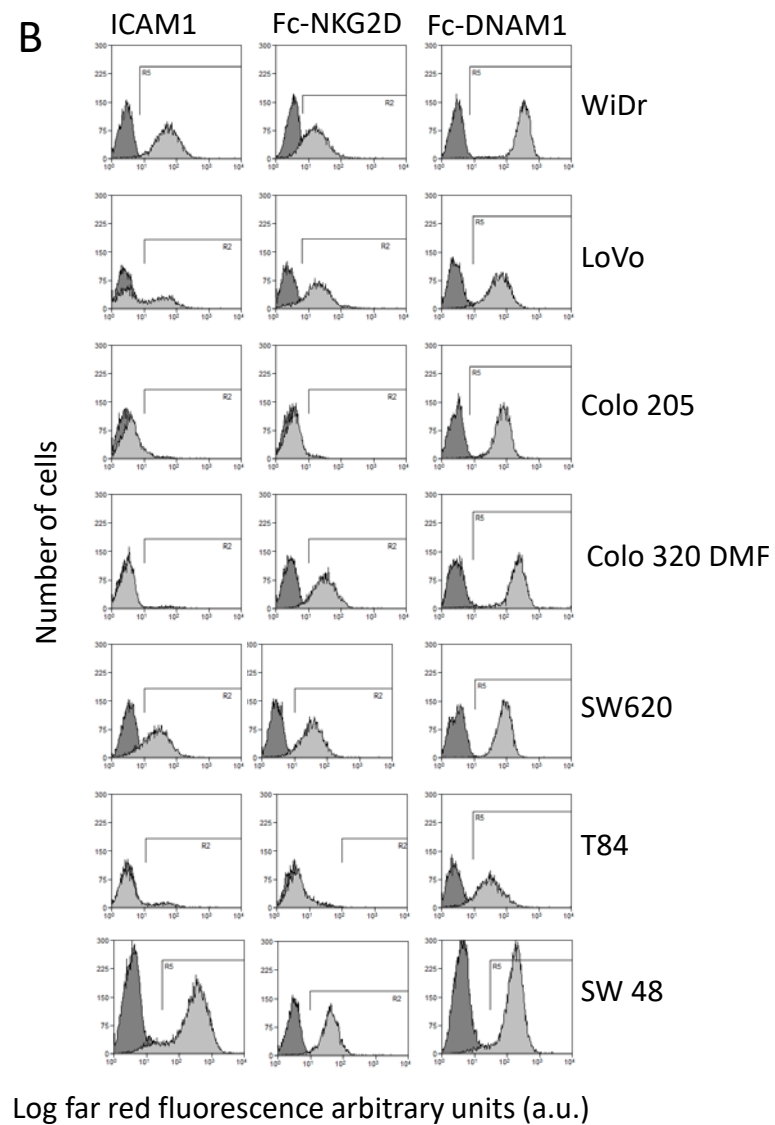

Supplement: Figure S2 — Expression of intercellular adhesion molecule (ICAM)1, NKG2D ligands (NKG2D-L) or DNAM1 ligands (DNAM1-L) on CRC cell lines. The carcinoma cell lines Caco2, HT29, HCT15, SW480, DLD1, HCT116, LS180 (A), WiDr, LoVo, Colo205, Colo320 DMF, SW620, T84, and SW480 (B) were analyzed for the expression of ICAM1, with the specific monoclonal antibodies, or NKG2D-L or DNAM1-L with the Fc-NKG2D or Fc-DNAM1 chimeric molecules by immunofluorescence assay and FACS analysis. In each panel, the negative control (AlexaFluor647 goat anti-mouse for ICAM1 and AlexaFluor647 human antiserum for the chimeras, black histograms) vs positive samples (gray histograms) is shown. Data are expressed as Log far-red fluorescence intensity (arbitrary units, a.u.) vs number of cells. [file Image_2.PDF]

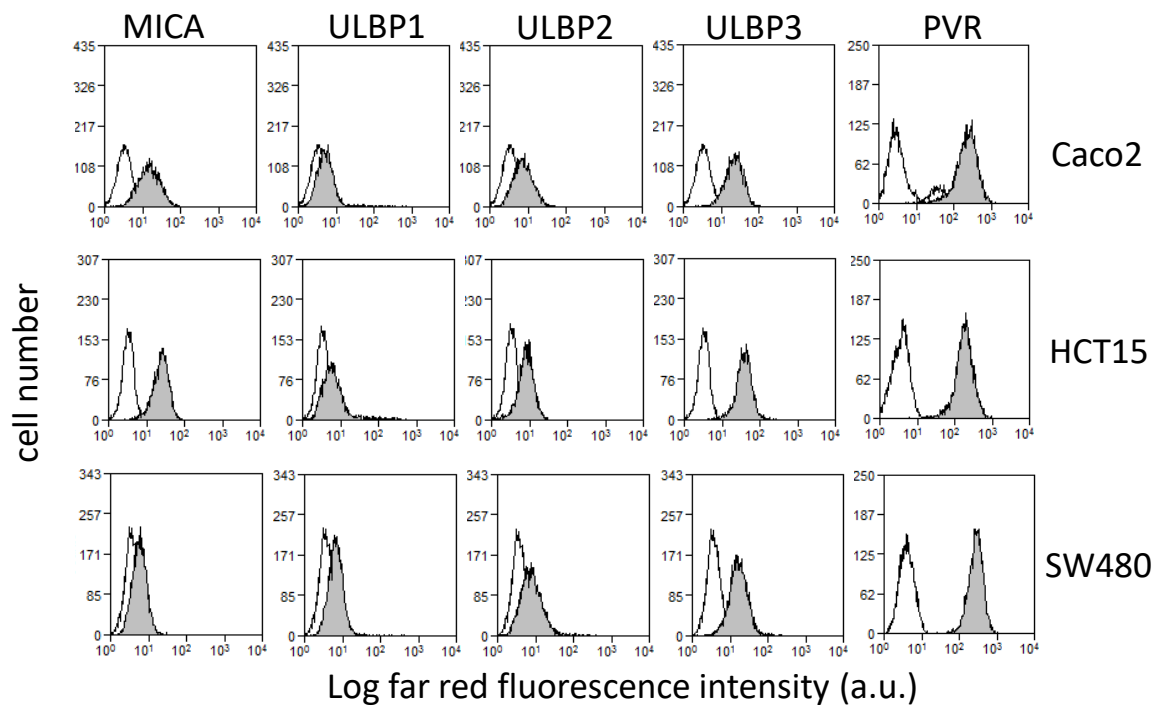

Supplement: Figure S3 — Expression of MICA, ULBPs, or poliovirus receptor (PVR) on selected CRC cell lines. The carcinoma cell lines Caco2, HCT15, and SW480 were analyzed for the expression of MICA, ULBP1, ULBP2, ULBP3, and PVR with specific monoclonal antibodies by immunofluorescence assay and FACS analysis. In each panel, the negative control (AlexaFluor647 goat anti-mouse, white histograms) vs positive samples (gray histograms) is shown. Data are expressed as Log far-red fluorescence intensity (arbitrary units, a.u.) vs number of cells. [file Image_3.PDF]

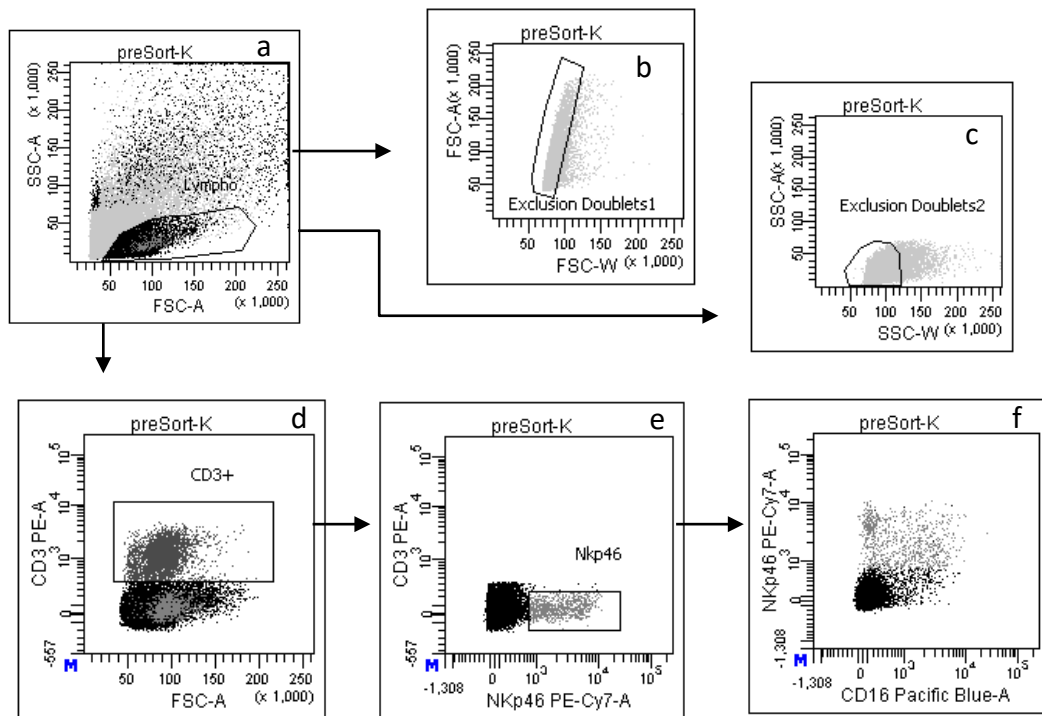

Costa et al. Suppl. Fig.4

Supplement: Figure S4 — Sorting strategy for NKp46+CD3− cells from CRC. NKP46+CD3− cell sorting from the OMCR16-030 CRC is shown as an example. Representative gating strategy: plots show first the recognition of the population of interest, without doublets, than the target of sorting NKp46+cells on CD3−. (A) Gray dots are doublet 1 and 2 events [depicted in panels (B,C)] excluded on the basis of physical parameters; (D) dark gray dots are cells excluded on the basis of CD3 expression. (E) Gray dots are sorted NKp46+CD3− cells. (F) CD16 and NKp46 expression (NKP46 PE-Cy7 vs CD16 Pacific Blue) on CD3− cells sorted in panel (E). [file Image_4.PDF]
